# Dosage of botulinum toxin in patients undergoing treatment for hemifacial spasm: is there modification during follow-up?

**DOI:** 10.1055/s-0044-1793935

**Published:** 2025-01-15

**Authors:** Fátima de Menezes Dantas, Felipe Olobardi Freire, Agábio Diógenes Pessoa Neto, Clécio de Oliveira Godeiro Júnior, Rodrigo Alencar e Silva

**Affiliations:** 1 Universidade Federal do Rio Grande do Norte, Centro de Ciências da Saúde, Natal RN, Brazil.; 2 Instituto de Ensino e Pesquisa Alberto Santos Dumont, Ambulatório Multiprofissional de Doença de Parkinson, Macaíba RN, Brazil.; 3 Universidade Federal do Rio Grande do Norte, Hospital Universitário Onofre Lopes, Serviço de Neurologia, Natal RN, Brazil.

**Keywords:** Hemifacial Spasm, Botulinum Toxins, Botulinum Toxins, Type A

## Abstract

**Background** The movement disorder known as hemifacial spasm is characterized by involuntary contractions of the muscles that are innervated by the facial nerve. The treatment of choice for this condition is botulinum toxin injections.

**Objective** To analyze the botulinum toxin dosage in patients undergoing treatment for hemifacial spasm during a 14-year period.

**Methods** A retrospective study of medical records from patients treated at the Neurology Service of Hospital Universitário Onofre Lopes, Universidade Federal do Rio Grande do Norte, from 2010 to 2024, was performed.

**Results** A total of 151 patients met the inclusion criteria. The dose of botulinum toxin revealed a statistically significant increase during the first 3.46 years of follow-up. In the long-term, a trend toward dose stabilization was identified. The median latency for the onset of effect was 4 days, while the median duration of effect was 3 months. All side effects were temporary, with the most common being hemifacial weakness (17.9%) and palpebral ptosis (3.3%). Most patients presented primary hemifacial spasm (88.1%), with a neurovascular conflict identified in 24.1% of cases.

**Conclusion** The increase in botulinum toxin dosage during the first years may be explained by dosage adjustment to control hemifacial spasm with the lowest possible doses. A prolonged interval between applications may also be associated with this increase. Dose stabilization tends to be achieved over time, indicating disease control.

## INTRODUCTION

Hemifacial spasm (HFS) is a disorder characterized by sporadic and involuntary contractions, predominantly unilateral, which affect the muscles innervated by the facial nerve. 1 2 3 Its pathophysiology is not fully known. In most cases, it appears to be related to the compression of the facial nerve at the exit point of the brainstem by an aberrant blood vessel. 4 5 6 7 Hemifacial spasm has an annual incidence of 0.78 per 100 thousand individuals and can lead to discomfort with one's appearance. 5 7 8 9 Without specific treatment, spontaneous regression is rare. 3 5 10

The treatment of choice for hemifacial spasm is botulinum toxin injections in the muscles affected. The toxin inhibits the release of acetylcholine in the neuromuscular junction, resulting in blocking of the muscle spasms. 3 5 8 11 Since the effect of botulinum toxin is temporary, 11 12 periodic injections are required to maintain its clinical results.

Despite the extensive experience with the use of botulinum toxin for control of HFS worldwide, few studies have been conducted with a large cohort of patients in Brazil. 13 In this context, this study's objective was to analyze the dosage of botulinum toxin in 151 patients from a tertiary service over a 14-year period.

## METHODS

### Patients

The sample of the present study consisted of patients treated at the Botulinum Toxin Outpatient Clinic of the Neurology Service at Hospital Universitário Onofre Lopes, Universidade Federal do Rio Grande do Norte, from January 2010 to January 2024.

The inclusion criteria were subjects with a clinical diagnosis of hemifacial spasm and age ≥ 18 years, and the exclusion criteria were patients submitted to > 4 botulinum toxin administrations during the study period and those with incomplete medical records.

Based on these criteria, out of an initial sample of 210 patients, 151 were included in the analysis. The study was approved by the Institutional Review Board at Hospital Universitário Onofre Lopes (CAAE: 72821023.2.0000.5292). All participants signed an informed consent declaration.

### Study design

The present is a retrospective study. Clinico-epidemiological and supplementary exam data were collected from medical records. Regarding the initial administration of botulinum toxin, the following data were collected: dosage, improvement in spasms, level of satisfaction following the first application, latency, duration, and adverse effects. For each subsequent application, the date and the dose used (in units) were recorded. The variation in dosage for each muscle was not analyzed; only the total dosage variation per patient was considered. To check the data and monitor response to treatment, patients were inquired about improvement in spasms, level of satisfaction following injection, latency, duration, and adverse effects at each new appointment.

### Treatments and data collection

**Figure 1:**
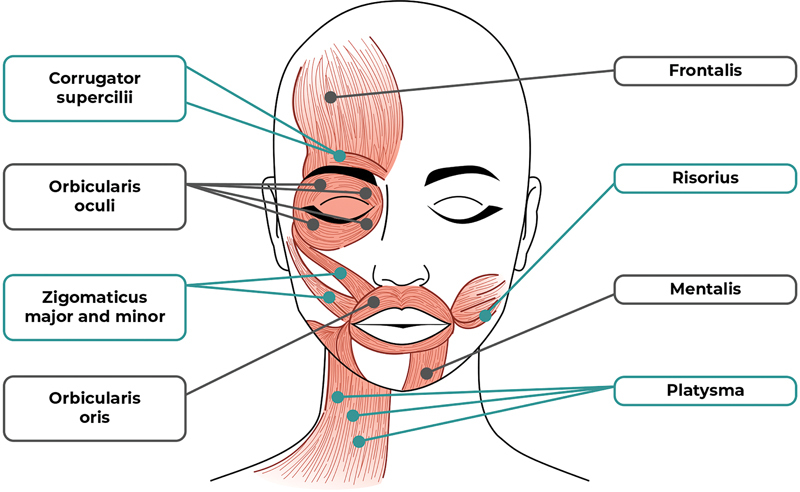
Botulinum toxin application points.

Patients received treatment with abobotulinumtoxinA (ABOtx) 500 U or onabotulinumtoxinA (ONAtx) 100 U. Every recommendation provided by the manufacturer has been strictly followed. For a concentration of 5 U per 0.1 mL, 2 mL of 0.9% saline solution was used to dilute a 100-U vial of ONAtx. Following diluting in 2.5 mL of 0.9% saline solution, the ABOtx vial gave a concentration of 20 U per 0.1 mL. Both formulations were stored in the original packaging at a temperature of 2°C to 8°C, respecting their specified expiration date. The injections were administered subcutaneously. 14 15 The frontalis, corrugator supercilii, orbicularis oculi, zygomaticus major and minor, risorius, mentalis, orbicularis oris, and platysma muscles were the ones targeted for injection ( Figure 1 ). Aesthetic points for symmetry were done in the corrugator supercilii, zygomaticus, risorius, and mentalis muscles.

Although some authors argue that ONAtx and ABOtx are different drugs, with no exact equivalence between their units, 7 16 others suggest that there is an approximate ONAtx:ABOtx equivalence, ranging from 1:1.5 to 1:6 units. 14 17 In our study, we adopted an ONAtx:ABOtx ratio of 1:4 for most patients, in accordance to the Clinical Protocols and Therapeutic Guidelines for Dystonias and Hemifacial Spasm published by the Brazilian Ministry of Health in 2017, 18 which describes the use of 1:3 and 1:4 ratios. The use of 1:4 and, rarely, 1:5 ratios aimed to enhance the effect of the toxin in cases of unsatisfactory results. 19 All ABOtx dosages were converted and registered in terms of ONAtx.

Due to the academic character of our service, some injections were administered by physicians in the second or third year of neurology residence, while others were performed by RAS or COGJ. While the third-year residents had months of experience in administering injections, the second-year residents were performing their first botulinum toxin injections. All residents were always supervised by RAS or COGJ.

The initial dosage was adjusted based on the severity of the hemifacial spasm observed during the physical examination, as assessed by supervising physicians according to their clinical experience. The patient's subjective response, clinical examination, and reported side effects were taken into consideration when determining the subsequent dose. The patient's level of satisfaction was assessed using an internally developed scale, allowing the patient to describe their experience with botulinum toxin treatment for spasm as *bad* , *regular* , *good* , or *great* .

The minimum interval between applications was 3 months. The maximum median interval between applications was 13 months. This prolonged interval was often attributed to missed appointments and the subsequent need for rescheduling. Given the limited number of resident physicians in our service relative to the high demand from patients, rescheduled appointments could result in months of waiting. Injections were not performed from March to September 2020 because of the coronavirus disease 2019 (COVID-19) epidemic.

### Statistical analysis

Data were collected in a Microsoft Excel (Microsoft Corp., Redmond, WA, USA) spreadsheet. Descriptive statistical analyses were performed using Jamovi software (open source) and R-Studio version 4.3.1 for Windows (R Foundation for Statistical Computing, Vienna, Austria). A mixed linear model (MLM) test was performed with the aim of understanding whether there was any relationship between the variables' dosage (units of ONAtx per application) and weeks (12-week intervals). Dosage and week variables were also correlated with the Pearson test. The clinico-epidemiological data of patients with primary and secondary hemifacial spasm were compared using the Chi-squared and Fisher's Exact tests. The results were considered significant if *p* < 0.05.

## RESULTS

Out of the initial sample of 210 patients, 30 were excluded due to incomplete medical records, while another 29 patients received less than 4 administrations of botulinum toxin (less than 1 year of treatment). The clinico-epidemiological data of the patients, compared based on the etiology of the spasm (primary or secondary), are presented in Table 1 .

**Table 1:** Clinico-epidemiological data of the patients with hemifacial spasm

		Primary HFS	Secondary HFS	*p* -value
		*N = 133 (88.1%)*	*N = 18 (11.9%)*	
**Gender**	Male	36 (27.1%)	5 (27.8%)	1.000 ^a^
	Female	97 (72.9%)	13 (72.2%)	
**Age of symptom onset, years**	10–19	2 (1.5%)	0 (0.0%)	0.530 ^b^
	20–29	2 (1.5%)	1 (5.6%)	
	30–39	16 (12.0%)	4 (22.2%)	
	40–49	28 (21.1%)	2 (11.1%)	
	50–59	49 (36.8%)	8 (44.4%)	
	60–69	28 (21.1%)	2 (11.1%)	
	70–79	7 (5.3%)	1 (5.6%)	
	80–89	1 (0.8%)	0 (0.0%)	
**Symptom onset site**	Upper face	96 (72.2%)	7 (38.9%)	0.009 ^b,c^
	Lower face	14 (10.5%)	6 (33.3%)	
	Both	23 (17.3%)	5 (27.8%)	
**Affected side**	Right	47 (35.3%)	5 (27.8%)	0.652 ^b^
	Left	85 (63.9%)	13 (72.2%)	
	Both	1 (0.8%)	0 (0.0%)	
**Family history of HFS**	Yes	8 (6.0%)	2 (11.1%)	0.493 ^b^
	No	123 (92.5%)	16 (88.9%)	
	Cannot report	2 (1.5%)	0 (0.0%)	
**Hypertension associated**	Yes	77 (57.9%)	8 (44.4%)	0.280 ^a^
**Etiology of HFS**	Neurovascular conflict	32 (24.1%)	0 (0.0%)	< 0.001 ^a,c^
	Peripheral facial paralysis	0 (0.0%)	17 (94.4%)	
	Abnormality of the superior fossa	0 (0.0%)	1 (5.6%)	
	Undetermined	101 (75.9%)	0 (0.0%)	
Complementary exams*	ENMG	10 (6.8%)	4 (16.8%)	0.150 ^a^
	CT scan	26 (17.7%)	2 (8.3%)	
	MRI	89 (60.5%)	12 (50.0%)	
	None	22 (15.0%)	6 (25.0%)	

Abbreviations: CT, computed tomography; ENMG, electroneuromyography; HFS, hemifacial spasm; MRI, magnetic resonance imaging.

Notes: *Some patients underwent more than one exam. Total number of exams = 171 (Primary HFS = 147; Secondary HFS = 24); ^a^ Fisher's Exact Test; ^b^ Chi-squared Test.

A total of 2,111 injections of botulinum toxin were administered, with an average of 14 applications per patient (standard deviation [SD] ± 6.93). Fifteen patients (9.93%) switched from ONAtx 100 U to ABOtx 500 U throughout the treatment ( Table 2 , which follows the model of a previous study 7 ). The main reasons for the switches were the same described in that study: unsatisfactory results with the injections, side effects and unavailability of a specific brand.

**Table 2:** Types and directions of shifts from onabotulinumtoxinA 100 U to abobotulinumtoxinA 500 U

Number of shifts	Direction	Number of cases
1	A ➔ B	3
	B ➔ A	3
2	A ➔ B ➔ A	6
	B ➔ A ➔ B	1
3	A ➔ B ➔ A ➔ B	1
4	A ➔ B ➔ A ➔ B ➔ A	1

Notes: A, onabotulinumtoxinA 100 U; B, abobotulinumtoxinA 500 U.

**Figure 2:**
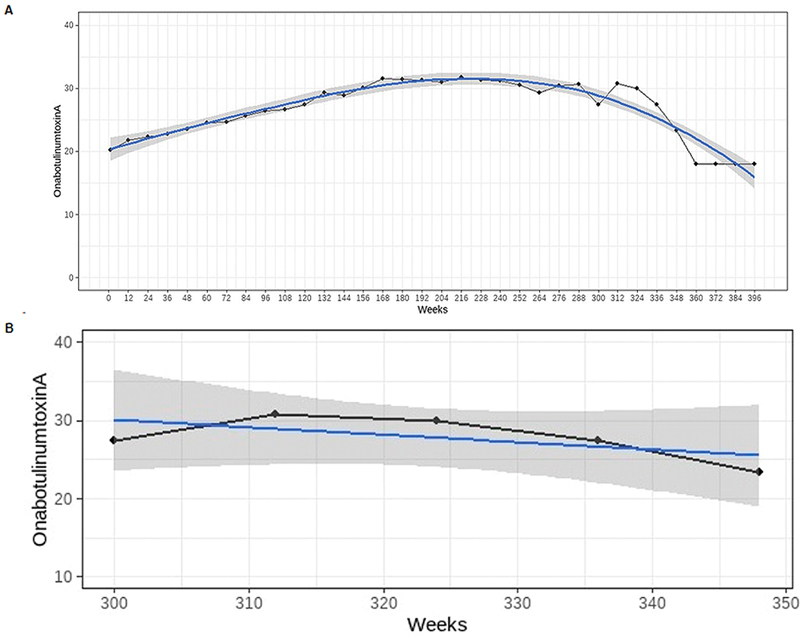
Notes: The blue line represents the trend of botulinum toxin dose variation. The shaded gray area represents the confidence interval. Dosage of onabotulinumtoxinA (units) over time (weeks), during the entire study period ( **A** ) and during weeks 300 to 348, a period when the confidence interval widens ( **B** ).

The average initial dose of ONAtx 100 U was 21.5 U (SD ± 9.00). The analysis of successive treatments revealed a trend of increasing the dose of ONAtx until week 180 ( *p* < 0.05), with a median increase of 0.046 units per week ( Figure 2 ). The median effect latency of the first administration was 4 days, and the median effect duration was 3 months. The results related to the first application of botulinum toxin are presented in Table 3 .

**Table 3:** Results of the first application of onabotulinumtoxinA

		Number of individuals	%
**Improvement of symptoms**	Yes	145	96
	No	6	4
**Level of satisfaction**	Bad	1	0.7
	Regular	21	13.9
	Good	81	53.6
	Great	48	31.8
**Side effects**	Palpebral ptosis	5	3.3
	Hemifacial weakness	27	17.9
	Significant hematoma	1	0.7
	Other	12	7.9
	None	105	69.5
	Allergic reaction, significant hematoma and other	1	0.7

## DISCUSSION

The analysis of botulinum toxin injection over time revealed a statistically significant increase in doses during the first 3.46 years of follow-up ( Figure 2A ). This finding is consistent with previous studies. 3 7 10 20 21 22 In fact, in a 10-year analysis of botulinum toxin treatment, Pérez-Saldaña et al. 23 found a significant increase in dosage during the first 4 years of treatment. Progressive dose increase could be explained by our practice of initially administering low doses and making gradual adjustments until hemifacial spasm is controlled, to use the lowest necessary amount and reduce the risk of side effects. This increase may also be justified by the intervals between doses, which are sometimes extended due to high demand in our service.

It is unlikely that this increase is related to the formation of neutralizing antibodies, since hemifacial spasm is one of the facial movement disorders treatable with the lowest rates of toxin resistance. 23 Furthermore, factors that increase the risk of antibody formation, such as short intervals between injections and the use of booster injections to achieve optimal efficacy, were not observed in our study. 20 However, it is important to mention that neither the frontalis test, which assesses resistance to botulinum toxin 24 nor biomolecular tests for antibody detection were performed.

After week 180 (3.46 years), our data showed a tendency toward dose stabilization ( Figure 2A ). Although from week 300 (5.75 years) on, the dose appeared to decrease over time, this trend was not statistically significant. Thus, we conclude that, in the long term, the dose of botulinum toxin stabilizes, which can be explained by achieving an optimized dose for the intensity of the patient's symptoms. There is no consensus in the literature regarding the long-term behavior of toxin dosage: some authors state that it remains constant, 19 23 while others argue that it increases 3 7 16 or decreases. 25

We attribute the apparent trend toward reduction to the small number of patients who had more than 5.75 years of treatment for hemifacial spasm. This resulted in a widening of the confidence interval of the analysis ( Figure 2B ). Therefore, it cannot be stated that the dose decreases over time, nor can it be assumed that muscle atrophy, or other phenomena associated with a reduction in toxin dosage, has occurred.

In this study, only 9.93% of the patients switched between botulinum toxin formulations. This suggests that most patients respond well to the initial formulation used, without the need to change brands over time. The latency and the duration of the toxin's effect were consistent with findings reported in other studies. 2 3 7 11

Clinico-epidemiological data from our sample characterized hemifacial spasm as a condition more common in women in their 5th decade of life, that predominantly affects the left side of the face and has few cases of family history. This description is similar to those of other case series. 1 2 3 7 20 26 27 Additionally, hypertension was present in the majority of our patients, suggesting a possible association between hemifacial spasm and high-pressure levels, already described by some authors. 28 29

According to our data, primary HFS was more commonly associated with the onset of symptoms in the upper segment of the face compared with secondary HFS ( *p* = 0.009). This is likely due to the involvement of different anatomical portions of the facial nerve depending on the etiology of the spasm. 30 In primary HFS, contractions typically begin in the orbicularis oculi muscle and subsequently spread to other muscles of the hemiface. 7 30 Our data also revealed similar percentages of onset of symptoms in the upper face (38.9%), lower face (33.3%), and both regions (27.8%) in secondary HFS, with no statistical difference. An Indian study 31 also found no statistical difference between the regions of spasm onset, although the upper face region was more frequent (75.36%), and the lower face less frequent (1.44%). We attribute our finding of more similar percentages between the regions to the small number of patients with secondary HFS in our sample.

Most patients presented with primary HFS (88.1%), a percentage similar to that reported in other studies. 1 2 3 32 A total of 101 patients (66.8%) underwent brain magnetic resonance imaging (MRI), although we did not find records indicating how many had MRI with angiography. Most of our patients (75.9%, *p* < 0.01) remained with the cause of the spasm undetermined. We attribute this to the absence of more specific tests capable of detecting vascular abnormalities, such as fast imaging employing steady state acquisition (FIESTA) with angiography, a sequence not typically included in routine brain MRI protocols. 33 34 Furthermore, high resolutions are recommended for identifying neurovascular changes, such as the 1.5-T or, preferably, the 3-T magnet. 35

Most patients reported improvement in their symptoms following the first application (96%), expressing a good or great level of satisfaction with the results (85.4%). Side effects were perceived by 30.5% of the patients, with hemifacial weakness (17.9%) and palpebral ptosis (3.3%) being the most common ( Table 3 ). Although these effects are listed among the most prevalent in various studies, their distribution is variable (hemifacial weakness ranges from 5.56 to 23%, while palpebral ptosis ranges from 3.2 to 36.11%). 3 7 11 32 Other side effects commonly referred to in the literature are lacrimation, hematoma, swelling, dry eye, diplopia, and lagophthalmos. 3 7 36 37 Only one case of allergic reaction was identified in our study, a rate similar to that reported in a randomized controlled trial. 38 All cases of side effects were temporary. Although these data are based on the subjective perception of patients, they suggest that botulinum toxin is a satisfactory and safe treatment for HFS, as demonstrated in previous studies. 2 3 7 20 32

One limitation of this study is the selection bias: as the patients were from a tertiary service, cases of HFS tend to be more severe, possibly requiring higher doses of botulinum toxin for control. Additionally, some patients were referred after receiving treatment at other services, with no record of previously used doses available for this analysis. There was also a 6-month application interruption due to the COVID-19 pandemic, which affected all patients. Furthermore, the equivalence adopted between ABOtx and ONAtx of 5:1 units is not exact, as the literature reveals differences in terms of outcomes and side effects between the two brands. 7 However, since only a small number of patients (0.71% of the injections) used ABOtx, we believe its influence on the dose analysis was minimal.

The use of aesthetic points and the conversion rate of ONAtx:ABOtx at 1:5 are limitations of our study, as they may have contributed to the increase in total dosage over time. Additionally, the variables of satisfaction level and improvement were assessed subjectively, without the use of a clinically validated scale. Although these variables, along with the presence of side effects, are evaluated at each new consultation, in this study, we only recorded responses after the first consultation, without follow-up analysis. Finally, the administration of botulinum toxin by physicians with varying levels of experience and the variation in intervals between applications are also limitations for analyzing dosage variation over time.

Based on our data, we conclude that it is important to continue treatment with botulinum toxin even if the symptoms are not fully controlled with the initial injections. An adjustment period of a few years is necessary to determine the optimal dose, after which the dosage tends to stabilize. To achieve this, we believe that the patient should have regular and long-term follow-up, with scheduled visits ideally every three months, to prevent the toxin from wearing off.

In summary, the present study demonstrated that the dose of botulinum toxin for treatment of HFS increases over the first years of treatment, stabilizing later. The increase in dosage may be due to the need for dose adjustments until an optimal level is reached, which allows dose stabilization. Despite the visualization of a long-term decrease in dose, this finding was not statistically significant. To more accurately assess long-term dosage modifications, prospective studies with larger patient cohorts are needed.

## References

[1] Felicio A C Godeiro-Junior C de O Borges V Silva S M Ferraz H B Clinical assessment of patients with primary and postparalytic hemifacial spasm: a retrospective study Arq Neuropsiquiatr 2007 65(3B):783 786 10.1590/s0004-282X2007000500009 17952280

[2] Pandey S Jain S Clinical features and response to botulinum toxin in primary and secondary hemifacial spasm Neurol India 2018 66 04 1036 1042 10.4103/0028-3886.236959 30038089

[3] Tambasco N Filidei M Nigro P Parnetti L Simoni S Botulinum Toxin for the Treatment of Hemifacial Spasm: An Update on Clinical Studies Toxins (Basel) 2021 13 12 881 10.3390/toxins13120881 34941718 PMC8706367

[4] Lefaucheur J P New insights into the pathophysiology of primary hemifacial spasm Neurochirurgie 2018 64 02 87 93 10.1016/j.neuchi.2017.12.004 29673579

[5] Duarte G S Rodrigues F B Castelão M Botulinum toxin type A therapy for hemifacial spasm Cochrane Database Syst Rev 2020 11 11 CD004899 10.1002/14651858.CD004899.pub3 33211908 PMC8078498

[6] Jariyakosol S Hirunwiwatkul P Lerdlum S Phumratprapin C Prevalence and Associated Factors of Neurovascular Contact in Patients With Hemifacial Spasm Asia Pac J Ophthalmol (Phila) 2015 4 04 212 215 10.1097/APO.0000000000000088 26176193

[7] Bentivoglio A R Fasano A Ialongo T Soleti F Lo Fermo S Albanese A Outcome predictors, efficacy and safety of Botox and Dysport in the long-term treatment of hemifacial spasm Eur J Neurol 2009 16 03 392 398 10.1111/j.1468-1331.2008.02507.x 19364366

[8] Cardoso Júnior J A Aspectos clínicos, demográficos e neurocomportamentais em pacientes com espasmo hemifacial [dissertation] Ribeirão Preto (SP) Faculdade de Medicina de Ribeirão Preto 2018

[9] Silva R A Desenvolvimento de modelo simulador facial para aquisição de habilidades manuais na técnica de aplicação de toxina botulínica no espasmo hemifacial e blefaroespasmo [dissertation] Natal (RN) Universidade Federal do Rio Grande do Norte, Natal 2022

[10] Barbosa E R Takada L T Gonçalves L R Costa R M Silveira-Moriyama L Chien H F Botulinum toxin type A in the treatment of hemifacial spasm: an 11-year experience Arq Neuropsiquiatr 2010 68 04 502 505 10.1590/s0004-282X2010000400006 20730300

[11] Batisti J P Kleinfelder A D Galli N B Moro A Munhoz R P Teive H A Treatment of hemifacial spasm with botulinum toxin type a: effective, long lasting and well tolerated Arq Neuropsiquiatr 2017 75 02 87 91 10.1590/0004-282X20160191 28226076

[12] Ledda C Artusi C A Tribolo A Time to onset and duration of botulinum toxin efficacy in movement disorders J Neurol 2022 269 07 3706 3712 10.1007/s00415-022-10995-2 35113259 PMC9217780

[13] Fowler F A Yabumoto C Osaki M H Profile of patients with essential blepharospasm and hemifacial spasm in the two largest ophthalmology reference centers in Brazil Arq Bras Oftalmol 2023 87 06 e20220160 10.5935/0004-2749.2022-0160 37851740 PMC11630479

[14] Dysport® (toxina botulínica A) [Internet] São Paulo Beaufour Ipsen Farmacêutica Ltda 2023[cited 2024 Aug. 23]. Available from:*https://consultas.anvisa.gov.br/#/bulario/q/?numeroRegistro=116370143*

[15] Botox® (toxina botulínica A) [Internet] São Paulo Allergan Produtos Farmacêuticos Ltda 2024[cited 2024 Aug. 23]. Available from:https://consultas.anvisa.gov.br/#/bulario/q/?nomeProduto=BOTOX

[16] Marchetti A Magar R Findley L Retrospective evaluation of the dose of Dysport and BOTOX in the management of cervical dystonia and blepharospasm: the REAL DOSE study Mov Disord 2005 20 08 937 944 10.1002/mds.20468Erratum in: Mov Disord. 2005 Aug;20(8):1089. Råuzizka, Evzen [corrected to Růzicka, Evzen]. PMID: 15810022 15810022

[17] Ozer I S Kuzu Kumcu M Tezcan Aydemir S Akbostanci M C Dose conversion ratio, comparative efficacy, and adverse events after switching from onabotulinum toxin A to abobotulinum toxin A for neurological conditions Clin Neurol Neurosurg 2021 209 106889 10.1016/j.clineuro.2021.106889 34461363

[18] Brazil. Ministry of Health. Health Care Secretariat. Ordinance n. 1. It approves the Clinical Protocol and Therapeutic Guidelines for Dystonias and Hemifacial Spasm, of May 29, 2017. Available from:https://www.gov.br/conitec/pt-br/midias/protocolos/protocolo_uso/pcdt_distonias_e_espasmo_hemifacial_29_05_2017.pdfAccessed 2024; Aug 23

[19] Scaglione F Conversion Ratio between Botox®, Dysport®, and Xeomin® in Clinical Practice Toxins (Basel) 2016 8 03 65 10.3390/toxins8030065 26959061 PMC4810210

[20] Ababneh O H Cetinkaya A Kulwin D R Long-term efficacy and safety of botulinum toxin A injections to treat blepharospasm and hemifacial spasm Clin Exp Ophthalmol 2014 42 03 254 261 10.1111/ceo.12165 23844601

[21] Ramirez-Castaneda J Jankovic J Long-term efficacy, safety, and side effect profile of botulinum toxin in dystonia: a 20-year follow-up Toxicon 2014 90 344 348 10.1016/j.toxicon.2014.07.009 25130293

[22] Gil Polo C Rodríguez Sanz M F Berrocal Izquierdo N Blepharospasm and hemifacial spasm: long-term treatment with botulinum toxin Neurologia 2013 28 03 131 136 10.1016/j.nrl.2012.03.009 22652139

[23] Pérez-Saldaña M T Parkhutik V Boscá-Blasco M E Claramonte B Burguera-Hernández J A Espasmo hemifacial: más de 10 años de tratamiento con toxina botulínica Rev Neurol 2007 45 10 582 586 18008262

[24] Ramos V F Karp B I Lungu C Alter K Hallett M Clinical Response to IncobotulinumtoxinA, after Demonstrated Loss of Clinical Response to OnabotulinumtoxinA and RimabotulininumtoxinB in a Patient with Musician's Dystonia Mov Disord Clin Pract 2014 1 04 383 385 10.1002/mdc3.12094 27066521 PMC4822507

[25] Echeverría Urabayen A Fanjul S Meseguer E García Ruiz Espiga P J Evolucion de la dosis de toxina botulinica tipo A en distonias craneocervicales. Estudio comparativo a lo largo de ocho años Rev Neurol 2004 38 06 511 513 15054712

[26] Wang L Hu X Dong H Clinical features and treatment status of hemifacial spasm in China Chin Med J (Engl) 2014 127 05 845 849 24571874

[27] Yaltho T C Jankovic J The many faces of hemifacial spasm: differential diagnosis of unilateral facial spasms Mov Disord 2011 26 09 1582 1592 10.1002/mds.23692 21469208

[28] Leong J L Li H H Chan L L Tan E K Revisiting the link between hypertension and hemifacial spasm Sci Rep 2016 6 21082 10.1038/srep21082 26891766 PMC4759578

[29] Xu F Gu P Yuan H Analysis of risk factors related to the progression rate of hemifacial spasm Front Neurol 2024 15 1.35728E6 10.3389/fneur.2024.1357280 PMC11007217 38606273

[30] Colosimo C Bologna M Lamberti S A comparative study of primary and secondary hemifacial spasm Arch Neurol 2006 63 03 441 444 10.1001/archneur.63.3.441 16533973

[31] Batla A Goyal C Shukla G Goyal V Srivastava A Behari M Hemifacial spasm: clinical characteristics of 321 Indian patients J Neurol 2012 259 08 1561 1565 10.1007/s00415-011-6376-3 22222858

[32] Herrero-Infante Y Rodríguez-Sanz A Máñez-Miró J Vivancos-Matellano F Hemifacial spasm through the last three decades: From etiology to efficacy and safety of long-term botulinum toxin treatment Clin Neurol Neurosurg 2021 203 106555 10.1016/j.clineuro.2021.106555 33662742

[33] Aktan D Depierreux F How to face the hemifacial spasm: challenges and misconceptions Acta Neurol Belg 2024 124 01 17 23 10.1007/s13760-023-02342-7 37498482

[34] Chen S R Neurological Imaging for Hemifacial Spasm Int Ophthalmol Clin 2018 58 01 97 109 10.1097/IIO.0000000000000212 29239882

[35] Hermier M Imaging of hemifacial spasm Neurochirurgie 2018 64 02 117 123 10.1016/j.neuchi.2018.01.005 29705020

[36] Yahalom G Janah A Rajz G Eichel R Therapeutic Approach to Botulinum Injections for Hemifacial Spasm, Synkinesis and Blepharospasm Toxins (Basel) 2022 14 05 362 10.3390/toxins14050362 35622608 PMC9147094

[37] Sorgun M H Yilmaz R Akin Y A Mercan F N Akbostanci M C Botulinum toxin injections for the treatment of hemifacial spasm over 16 years J Clin Neurosci 2015 22 08 1319 1325 10.1016/j.jocn.2015.02.032 26100157

[38] Li Y J Huang Y Ding Q Gu Z H Pan X L Evaluation of concentrations of botulinum toxin A for the treatment of hemifacial spasm: a randomized double-blind crossover trial Genet Mol Res 2015 14 01 1136 1144 10.4238/2015.February.6.17 25730053

